# Relationships between adiponectin levels, the metabolic syndrome, and type 2 diabetes: a literature review

**DOI:** 10.1590/2359-3997000000316

**Published:** 2017-12-01

**Authors:** Anize Delfino von Frankenberg, André F. Reis, Fernando Gerchman

**Affiliations:** 1 Universidade Federal do Rio Grande do Sul Faculdade de Medicina Porto Alegre RS Brasil Programa de Pós-Graduação em Endocrinología, Faculdade de Medicina, Universidade Federal do Rio Grande do Sul (UFRGS), Porto Alegre, RS, Brasil; 2 Universidade Federal de Ciências da Saúde de Porto Alegre Departamento de Nutrição Porto Alegre RS Brasil Departamento de Nutrição, Universidade Federal de Ciências da Saúde de Porto Alegre (UFCSPA), Porto Alegre, RS, Brasil; 3 Universidade Federal de São Paulo Departamento de Medicina São Paulo SP Brasil Universidade Federal de São Paulo (Unifesp), Departamento de Medicina, Disciplina de Endocrinologia, São Paulo, SP, Brasil; 4 Hospital de Clínicas de Porto Alegre (HCPA) Porto Alegre RS Brasil Unidade de Metabolismo, Divisão de Endocrinologia, Hospital de Clínicas de Porto Alegre (HCPA), Porto Alegre, RS, Brasil

**Keywords:** Adiponectin, metabolic syndrome, type 2 diabetes

## Abstract

Elevated hepatic glucose production, impaired insulin secretion, and insulin resistance - abnormalities of glucose metabolism typically found in subjects with obesity - are major factors underlying the pathogenesis of type 2 diabetes (DM2) and the metabolic syndrome (MS). Adiponectin is a major regulator of glucose and lipid homeostasis via its insulin-sensitizing properties, and lower levels seems to be associated with the development of DM2 and MS. The purpose of this review is to clarify the mechanisms whereby adiponectin relates to the development of DM2 and MS and the association between polymorphisms of the adiponectin gene, circulating levels of the hormone, and its relationships with DM2. In addition, the impact of dietary lipids in the circulating levels of adiponectin will be addressed. According to the literature, circulating adiponectin levels seem to decrease as the number of MS components increases. Lower adiponectin concentrations are associated with higher intra-abdominal fat content. Therefore, adiponectin could link intra-abdominal fat with insulin resistance and development of MS. Therapeutic strategies that target the MS and its components, such as lifestyle modification through physical activity and weight loss, have been shown to increase adiponectin concentrations. Possible roles of diets containing either low or high amounts of fat, or different types of fat, have been analyzed in several studies, with heterogeneous results. Supplementation with n-3 PUFA modestly increases adiponectin levels, whereas conjugated linoleic acid supplementation appears to reduce concentrations when compared with unsaturated fatty acid supplementation used as an active placebo.

## INTRODUCTION

The prevalence of the metabolic syndrome (MS) and diabetes mellitus type 2 (DM2) in the North American population is 22.9% and 9.3%, respectively, according to NHANES and CDC data (1,2). Very similar prevalence figures have been reported in Brazil (3). In a recent systematic review of cross-sectional studies, the weighted average prevalence of MS was 29.8%, 20.1%, and 41.5% in the adult Brazilian population in urban, rural, and indigenous areas, respectively (3). According to the results of a study conducted with fixed-shift industry workers in the state of Rio Grande do Sul, being female, being older, and having sleep deprivation proved to be potential risk factors for MS, while having a higher education and eating a greater number of meals per day were considered protective factors against MS (4). Regarding DM2, the Multicenter Study on the Prevalence of Diabetes in Brazil estimated its prevalence the adult population at 7.6% in the late 1980s (5). According to 2012 data, 7.4% of respondents to a telephone survey representative of the entire adult population of all 26 state capitals and the Federal District reported a medical diagnosis of DM2 (6). Complications from DM2 result in high morbidity and mortality and an average 6-year reduction in average life expectancy when the disease is diagnosed at age 50 (7). The direct and indirect costs of DM2 amount to US$ 245 billion a year in the United States alone. Health expenditures on individuals with DM2 are increased twofold when compared to spending on individuals without the disease (1). In Brazil, DM is also considered a major public health problem. Its estimated cost per capita is US$ 1527.6/year; considering that an estimated 11.6 million Brazilians aged 20 to 79 have DM2, direct expenses related to this condition amount to approximately US$ 17 billion a year (8).

Obesity is associated with development of the MS, which is characterized by a cluster of risk factors for cardiovascular disease and DM2, such as hyperglycemia, elevated blood pressure, elevated triglycerides, low HDL cholesterol, and central obesity (9). Moreover, obesity and abdominal fat deposition cause a number of metabolic abnormalities that result in increased hepatic glucose output and decreased insulin sensitivity in skeletal muscle, liver, and adipose tissue - processes that are closely related to the pathogenesis of DM2 (10).

In view of the foregoing, the present review sought to clarify the mechanisms whereby the hormone adiponectin relates to the development of MS and DM2. The association between polymorphisms of the adiponectin gene, circulating levels of the hormone it encodes, and its relationships with DM2 will also be explored. In addition, the impact of diets with different levels and types of lipids on circulating levels of adiponectin will be addressed.

## ADIPONECTIN AND GLUCOSE METABOLISM

Adiponectin, a hormone present mainly in adipose tissue, is encoded by the *APM1* gene (chromosome 3q27). In humans, adiponectin plasma levels range from 3 to 30 μg/mL, which is among the highest plasma concentrations of a circulating protein. The adiponectin molecule is a 247-amino acid polypeptide and is secreted into circulation in three oligomeric isoforms: a low- molecular-weight trimer, an intermediate-molecular- weight hexamer and a high-molecular-weight complex (11). Some studies suggest that the high-molecular- weight isoform is most biologically active, and that lower levels of this form are associated with DM and coronary artery disease (12-14). Adiponectin acts through two receptors, AdipoR1 and AdipoR2; the former is expressed at higher levels in muscle tissue, and the latter, in liver tissue. Studies have demonstrated that the AdipoR1 receptor is also present in endothelial cells (15), cardiomyocytes (16), and pancreatic beta cells (17), while AdipoR2 is present in endothelial cells (18), and both receptors are present in the hypothalamus (19). Resistance to the action of insulin resulting from obesity causes downregulation of adiponectin receptors in muscle and liver (20). Furthermore, adiponectin expression blunts increases in insulin, TNF-α, endothelin-1, and glucocorticoids, factors implicated in the pathogenesis of insulin resistance, subclinical inflammation, endothelial dysfunction, and regulation of energy metabolism, and closely related to the development of MS, DM2, and cardiovascular disease (21). Accordingly, extensive research has shown that adiponectin levels are reduced in obesity (22,23), DM2 (22,24), and coronary artery disease (25-27).

To test the *in vivo* effect of adiponectin on insulin sensitivity, a lipoatrophy mouse model with adiponectin deficiency was developed. In these animals, replacement of physiological doses of adiponectin improved insulin resistance (28). Adiponectin stimulated fatty acid oxidation in muscle by increasing the expression of molecules involved in the transport of fatty acids (CD36), their combustion (acetyl coenzyme A oxidase), and dissipation of energy through increased expression of type 2 uncoupling protein (UCP-2) (28). Adiponectin replacement in these animals increased PPAR-alpha expression, fatty acid oxidation, and energy consumption, causing a reduction of triglyceride levels in muscle and liver tissue (28). The reduction in triglyceride content in skeletal muscle was associated with increased translocation of GLUT-4, which led to improved insulin sensitivity (28). In another study by the same group, acute treatment (up to 6 hours) of C2C12 myoblasts with adiponectin increased the oxidation of fatty acids and stimulated glucose uptake via activation of AMPK (29), leading to a reduction in levels of enzymes that indicate hepatic gluconeogenesis. Furthermore, wild-type mice that received a diet rich in total lipids had a reduction in adiponectin levels compared to a diet rich in carbohydrates. Adiponectin replacement in these animals also improved insulin resistance and hypertriglyceridemia induced specifically by the high-fat diet (28). Additionally, studies demonstrated in wild-type and *ob/ob* or streptozotocin- induced DM1 mouse models that an acute increase in circulating levels of adiponectin leads to a transient decrease in baseline glucose level by inhibiting enzymes involved in hepatic gluconeogenesis and hepatic glucose production rate (30,31). Based on these studies, it was demonstrated that stimulation of fatty acid oxidation in muscle and liver, increasing glucose uptake in skeletal muscle and suppressing hepatic gluconeogenesis, are potential routes through which adiponectin regulates insulin sensitivity (28,30,31). These data suggest that insulin resistance associated with a high-fat diet and obesity are partly related to a reduction in circulating adiponectin levels and that an increase of these levels would protect against the development of different components of MS, especially those related to the modulation of insulin sensitivity, body fat distribution, and lipoprotein metabolism (28).

Studies have suggested a relationship between adipokines, such as letptin and resistin, and DM-related vascular complications (32,33). Chronic kidney disease is considered a long-term complication of DM, and its development has been associated with higher levels of these adipokines (34). According to an experimental study, adipokines can lead to kidney injury by regulation of endothelial dysfunction, oxidative stress, and inflammatory processes (35). A longitudinal study of 161 subjects with diabetes followed from 2002 to 2013 demonstrated that plasma adiponectin increased in patients with renal insufficiency, and that its levels were positively associated with albuminuria (36). It is interesting to highlight that, in the context of chronic kidney disease, higher levels of adiponectin have been found to predict progression to end-stage renal disease (ESRD), cardiovascular mortality, and total mortality (37,38). Adiponectin levels are known to increase with decreasing glomerular filtration rate in chronic kidney disease, as a reflection of decreased renal clearance (39). As a result, ESRD features increased adiponectin levels and AdipoR1 expression (40). Both mechanisms may explain the association between higher adiponectin levels and total and cardiovascular mortality in this scenario.

## ASSOCIATION BETWEEN ADIPONECTIN GENE POLYMORPHISMS, CIRCULATING ADIPONECTIN LEVELS, AND DM2

Epidemiological studies have shown that DM2 clusters in families, suggesting a genetic contribution to its development. The cumulative risk of DM2 at age 65 was 14.8% for individuals with no family history of DM2, 22% for individuals with only one parent with DM2, and 41% for individuals whose parents are both affected by the disease (41).

Recent studies have shown that a number of genetic polymorphisms are associated with the development of obesity and DM2 (42,43). Genes that modulate the metabolism of adipose tissue and, consequently, are involved in the fatty acid synthesis and metabolism pathways are important determinants of body fat distribution and insulin sensitivity, which, in turn, are also related to abnormalities in glucose metabolism and development of DM2. Genetic variants of the adiponectin gene have also been associated with resistance to insulin action and DM2 (44).

It is estimated that 39-46% of the variability of circulating adiponectin levels is due to genetic factors (45,46). In this regard, a recent systematic review and meta-analysis compiled data from seven studies that explored the association between three single-nucleotide polymorphisms (SNPs), −11391G→A, +45T→G, and +276G→T, and plasma level of adiponectin (25). The −11391G→A SNP was associated with higher levels of circulating adiponectin in subjects carrying the A allele compared to subjects carrying only the G allele. No association was found between the +45T→G SNP and adiponectin levels. Regarding the SNP +276G→T, adiponectin levels showed a progressive increase in homozygotes for the G allele when compared to heterozygotes and homozygotes for the T allele (25).

Associations between adiponectin gene polymorphisms and risk of DM2 have also been widely explored in the literature. Among the nine chromosomal regions related to DM2, three (3q, 15q, and 20q) are found in various ethnic groups, such as the Japanese, German, and French (47). Interestingly, the 3q27 region containing the adiponectin gene once again suggests a role of adiponectin as a determinant of susceptibility to DM2. The 276 SNP in intron 2 (G vs. T) has been associated with distinct phenotypes of adiponectin levels, insulin resistance, and susceptibility to DM2. Individuals with the G/G genotype at position 267 had lower adiponectin levels and increased DM2 risk compared with T/T genotype carriers (24). Similar associations between the adiponectin gene and susceptibility to DM2 have also been demonstrated in German and French populations (48,49).

Given the large number of studies that have sought associations between different polymorphisms of the adiponectin gene and DM2, a recent systematic review and meta-analysis pooled the results of more than 2,000 individuals with DM2 vs. controls for four SNPs: −11391G→A, −11377C→G, +45T→G, and +276G>T (25). No association was demonstrated between the evaluated SNPs and risk of DM2. Subsequently, another systematic review and metaanalysis of 45 studies (9,986 individuals with DM2 vs. 16,222 control subjects) only assessed polymorphism +45T→G and, through a subgroup analysis, found an association between +45T→G and risk of DM2 in studies involving Asians. However, there was no such association in studies involving Caucasians (50). Regarding insulin resistance, an association between the +276G→T SNP and insulin resistance estimated by HOMA-IR has been observed. Resistance to insulin action was higher in individuals homozygous for the G allele compared to heterozygotes and those homozygous for the T allele, indicating higher insulin sensitivity in individuals carrying the T allele - the same allele that showed a trend toward association with higher levels of adiponectin (25).

In summary, studies have suggested that genetic factors modulate circulating adiponectin levels (27). Additionally, adiponectin gene variants have been associated with higher risk of developing DM2 and MS, especially in phenotypes associated with insulin resistance (51). However, this finding remains controversial.

## ADIPONECTIN AND THE METABOLIC SYNDROME

Studies have suggested that expression of the *APM1* gene in visceral abdominal fat is lower than in subcutaneous abdominal fat (52,53). This gene expression in adipose tissue correlates significantly with plasma levels, being higher in lean individuals and those with higher sensitivity to insulin action (23). Furthermore, the lowest concentration of adiponectin is associated more strongly with quantification of visceral abdominal fat than with subcutaneous abdominal fat, suggesting a possible relationship with MS (54).

**Figure 1:**
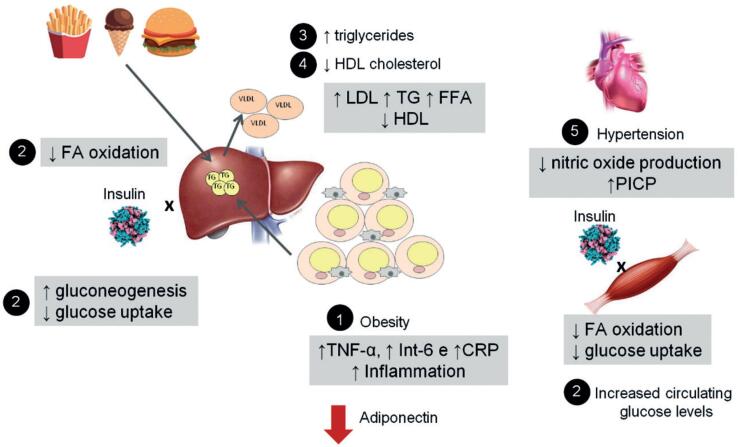
Mechanisms whereby a reduction in adiponectin levels is associated with the development of MS. **1:** Accumulation of visceral fat reduces production of adiponectin. Tissue inflammation increases levels of C-reactive protein (CRP) and inflammatory cytokines (TNF-α and interleukin-6), activating hepatic gluconeogenesis. **2:** Hepatic gluconeogenesis is activated and insulin sensitivity in muscle and liver is further reduced, resulting in increased circulating glucose levels. **3** and **4:** Reduction of triglyceride oxidation from adipose tissue and dietary lipids by the liver increases levels of free fatty acids (FFA) and production of VLDL, generating an imbalance in lipid profile (increased LDL cholesterol, triglycerides [TG], and reduced HDL cholesterol). **5:** Increased serum level of procollagen type I carboxy-terminal propeptide (PICP) intensifies arterial stiffness, and reduced nitric oxide production contributes to reduced vasodilation. These mechanisms, along with the pro-inflammatory environment, promote changes in blood pressure homeostasis, which contribute to the development of systemic arterial hypertension.

The inverse relation between adiponectin levels and criteria for MS has been described in the literature (47,55-58). It is well demonstrated that overweight individuals have lower levels of adiponectin compared to lean individuals, and that levels of this hormone decrease as BMI increases in men and women (55). In addition, higher levels of adiponectin were associated with a lower incidence of DM2 in a Japanese cohort followed for 5 years in order to better understand the factors related to development of DM. Individuals in the lowest tertile of adiponectin levels developed approximately nine times more DM2 than those individuals belonging to the highest tertile (56). Additionally, individuals with lower plasma levels of adiponectin have LDL- cholesterol molecules of smaller size, lower lipoprotein lipase activity, lower HDL-cholesterol levels, and higher triglyceride levels (47,58). Regarding blood pressure, lower levels of circulating adiponectin were observed in hypertensive compared to non-hypertensive patients, even after adjusting for obesity, insulin resistance, and DM2 (57). Studies have suggested an effect of adiponectin on blood pressure homeostasis. An increase in collagen deposition promoted by increased serum levels of procollagen type I carboxy-terminal propeptide (PICP) is associated with an acceleration of the arterial stiffening process, a phenomenon closely related to the development of hypertension (59) and MS (60). A cross-sectional study of 188 hypertensive patients without DM2 showed that higher adiponectin levels were associated with lower circulating levels of PICP (61). Reinforcing this hypothesis, lower levels of adiponectin were associated with increased arterial wall stiffness in a cohort of elderly individuals (62). An effect of adiponectin on endothelial function has also been demonstrated. Adiponectin increases gene expression and activates endothelial nitric oxide synthase through activation of AMPK (63), stimulating the synthesis of nitric oxide, an important endothelial factor and potent vasodilator (64). Additionally, it is known that the renin-angiotensin system plays an important role in regulating blood pressure and that, when activated, it perpetuates the inflammatory process in the arterial wall, increasing oxidative stress and fostering development of atherosclerosis (65,66). Through its antioxidant and anti-inflammatory effects, adiponectin inhibits the deleterious vascular effect of renin-angiotensin system activation and is closely related to dysregulation of blood pressure homeostasis in MS (67). Figure 1 shows the different mechanisms involved in the pathogenesis of MS related to hypoadiponectinemia.

Cross-sectional studies have evaluated the associations between the different MS components and adiponectin levels in populations with different metabolic profiles (68-70). The results of a crosssectional study conducted in the elderly U.S. Rancho Bernardo cohort demonstrated that individuals with MS had lower circulating levels of adiponectin compared to individuals without MS (68). Furthermore, the presence of each of the MS components was associated with lower levels of adiponectin (68). Koh and cols. included only Asian individuals over the age of 40, and found that lower adiponectin levels were associated with greater waist circumference and increased levels of triglycerides, CRP, fasting glucose, and insulin. Furthermore, individuals with higher circulating adiponectin had higher HDL-cholesterol levels (69). In a study conducted by von Frankenberg and cols. in Brazil, individuals were referred to a tertiary care hospital (*Hospital de Clínicas de Porto Alegre*, state of Rio Grande do Sul) for screening and evaluation of glucose metabolism abnormalities and MS. A replication analysis was performed in subjects undergoing cardiac catheterization at another tertiary referral center (*Hospital São Paulo*, in the city of São Paulo). This study demonstrated that levels of total and high-molecular-weight adiponectin were lower in the presence of MS, and were reduced with each increase in the number of components of MS. Adiponectin levels were mainly determined by their relation with HDL cholesterol, triglycerides, and waist circumference. In addition, blood glucose, subclinical inflammation, and insulin resistance partially explained why adiponectin levels were lower in individuals with compared to individuals without MS (70).

## EFFECT OF DIETARY LIPIDS ON CIRCULATING ADIPONECTIN LEVELS

Intervention studies have shown that adiponectin levels can be partly determined by different types of diet. Given the important role of adiponectin in carbohydrate and lipid metabolism, including improving insulin sensitivity and increasing fatty acid oxidation, diets that modify the quantity and quality of lipids ingested can have an impact on the metabolism and plasma levels of adiponectin (71,72). Several studies conducted in humans aimed to show the effect of diets with high or low levels of total lipids in the regulation of adiponectin (73-75). In a randomized clinical trial comparing a fat- restricted hypocaloric diet (27% fat, 52% carbohydrates, and 21% protein) vs. a high-fat diet (41% fat, 39% carbohydrates, and 20% protein), there were no changes in adiponectin levels observed at the end of 10 weeks of intervention (73). However, another study that compared a normal-lipid hypocaloric diet (30% fat) vs. a high-lipid (61% fat) diet showed 30% and 18% increases in adiponectin levels after 52 weeks of intervention respectively (75). However, providing isocaloric diets for weight maintenance with high fat (55% fat, 27% carbohydrates, 18% protein) or low fat (20% fat, 62% carbohydrates, 18 % protein) was not associated with differences in adiponectin levels after 4 weeks of intervention (74). These results suggest there is no consensus about the effect of total dietary lipid intake (low fat vs. high fat) on adiponectin levels in interventional studies conducted in humans.

Greater adherence to the Mediterranean style diet, which is rich in unsaturated fats, has been associated with higher adiponectin levels (76). This relationship is possibly attributable not only to the low glycemic load and moderate alcohol consumption characteristic of this diet, but also to its high content of oilseeds and olive and fish oil, which are dietary sources of polyunsaturated fatty acids (71). The mechanisms through which the Mediterranean diet has impacts on circulating levels of adiponectin is still unknown, but several hypotheses have been raised. The omega-3 type polyunsaturated fatty acids (n-3 PUFA) found in this diet can modulate adiponectin levels by interacting with the peroxisome proliferator-activated receptors alpha (PPAR-α) and gamma (PPAR-γ) (77). Activation of PPAR-α stimulated by consumption of n-3 PUFA increases expression of AdipoR1 and AdipoR2 in muscle and liver, improving sensitivity to this hormone in these tissues (78). Adiponectin then acts by reducing inflammation and oxidative stress, which ultimately leads to improved insulin sensitivity (79). Moreover, n-3 PUFAs activate PPAR-γ, thus increasing adiponectin levels through a second route. In an experimental study, consumption of n-3 PUFAs was associated with a twofold increase in expression of the adiponectin gene, which occurred parallel to a twofold to threefold increase in expression of the gene which encodes PPAR-γ (77). Thus, the activation of PPAR-α and PPAR-γ promoted by n-3 PUFAs increases adiponectin levels and activity, which results in improvement in obesity-induced inflammation and insulin resistance (78).

In addition to the n-3 PUFAs, other types of lipids have shown effectiveness in the regulation of adiponectin, among which conjugated linoleic acid (CLA) stands out. CLA can cause resistance to insulin action by reducing plasma levels of adiponectin (80). The mRNA levels of adiponectin were reduced after CLA supplementation in rats (81) and in cultured human adipocyte cells (82). Since the adiponectin gene is modulated by activation of PPAR-γ, suppression of the adiponectin gene can be partly attributed to the antagonistic effect of the trans-10, cis-12 CLA on PPAR-γ (83).

Analysis of the results of clinical trials conducted across different ethnic groups, genders, metabolic profiles, and diseases provides an understanding of the effects of lipid intake on circulating levels of adiponectin. In this regard, a recent systematic review and meta-analysis performed by our group showed that, in intervention studies comparing low-fat vs. high-fat diets, there was no association of total amount of fat with circulating levels of adiponectin (72). Omega-3 PUFA supplementation modestly increased circulating concentrations of adiponectin; however, these findings should be interpreted with caution, since publication bias was identified in this meta-analysis (72). However, CLA supplementation reduced adiponectin concentrations as compared with unsaturated fatty acid supplementation as active placebo (72).

## CONCLUSIONS

Circulating levels of adiponectin are reduced in the presence of the MS, cardiovascular disease, and DM2, and also decrease as the number of MS components increases. The association of adiponectin with HDL cholesterol, triglycerides, and abdominal fat may partly explain the lower levels of adiponectin found in individuals with MS. Among dietary interventions, diets low in total lipids have shown no effect on circulating adiponectin. Supplementation with n-3 polyunsaturated fatty acids, however, was associated with a moderate increase in adiponectin. In contrast, conjugated linoleic acid appears to reduce adiponectin levels when compared to unsaturated fat supplementation.

## References

[1] CDC. National Diabetes Statistics Report: Estimates of Diabetes and Its Burden in the United States. In: Services UDoHaH, editor. Atlanta; 2014.

[2] Beltran-Sanchez H, Harhay MO, Harhay MM, McElligott S. Prevalence and trends of metabolic syndrome in the adult U.S. population, 1999-2010. J Am Coll Cardiol. 2013;62(8):697-703. 10.1016/j.jacc.2013.05.064 PMC3756561 23810877

[3] de Carvalho Vidigal F, Bressan J, Babio N, Salas-Salvado J. Prevalence of metabolic syndrome in Brazilian adults: a systematic review. BMC Public Health. 2013;13:1198. 10.1186/1471-2458-13-1198 PMC3878341 24350922

[4] Canuto R, Pattussi MP, Macagnan JB, Henn RL, Olinto MT Metabolic syndrome in fixed-shift workers. Rev. Saúde Pública. 2015;49:30. 10.1590/S0034-8910.2015049005524 PMC4544368 26061455

[5] Malerbi DA, Franco LJ. Multicenter study of the prevalence of diabetes mellitus and impaired glucose tolerance in the urban Brazilian population aged 30-69 yr. The Brazilian Cooperative Group on the Study of Diabetes Prevalence. Diabetes Care. 1992;15(11):1509-16. 10.2337/diacare.15.11.1509 1468278

[6] Brasil V. Vigilância de fatores de risco e proteção para doenças crônicas por inquérito telefônico. In: Saúde Md, editor. 2012.

[7] Rao Kondapally Seshasai S, Kaptoge S, Thompson A, Di Angelantonio E, Gao P, Sarwar N, et al. Emerging Risk Factors Collaboration et al. Diabetes mellitus, fasting glucose, and risk of cause-specific death. N Engl J Med. 2011;364(9):829-41. 10.1056/NEJMoa1008862 PMC4109980 21366474

[8] IDF 2010 [cited Jun 22 2015th], Available from: http://www.diabetesatlas.org. Access on: Oct 26, 2017.

[9] Malik S, Wong ND, Franklin SS, Kamath TV, L'Italien GJ, Pio JR, et al. Impact of the metabolic syndrome on mortality from coronary heart disease, cardiovascular disease, and all causes in United States adults. Circulation. 2004;110(10):1245-50. 10.1161/01.CIR.0000140677.20606.0E 15326067

[10] Kahn SE, Hull RL, Utzschneider KM. Mechanisms linking obesity to insulin resistance and type 2 diabetes. Nature. 2006;444(7121):840-6. 10.1038/nature05482 17167471

[11] Pajvani UB, Du X, Combs TP, Berg AH, Rajala MW, Schulthess T, et al. Structure-function studies of the adipocyte-secreted hormone Acrp30/adiponectin. Implications fpr metabolic regulation and bioactivity. J Biol Chem. 2003;278(11):9073-85. 10.1074/jbc.M207198200 12496257

[12] Aso Y, Yamamoto R, Wakabayashi S, Uchida T, Takayanagi K, Takebayashi K, et al. Comparison of serum high-molecular weight (HMW) adiponectin with total adiponectin concentrations in type 2 diabetic patients with coronary artery disease using a novel enzyme-linked immunosorbent assay to detect HMW adiponectin. Diabetes. 2006;55(7):1954-60. 10.2337/db05-1525 16804063

[13] Kobayashi H, Ouchi N, Kihara S, Walsh K, Kumada M, Abe Y, et al. Selective suppression of endothelial cell apoptosis by the high molecular weight form of adiponectin. Circ Res. 2004;94(4):e27-31. 10.1161/01.RES.0000119921.86460.37 PMC4374479 14752031

[14] Pajvani UB, Hawkins M, Combs TP, Rajala MW, Doebber T, Berger JP, et al. Complex distribution, not absolute amount of adiponectin, correlates with thiazolidinedione-mediated improvement in insulin sensitivity. J Biol Chem. 2004;279(13):12152-62. 10.1074/jbc.M311113200 14699128

[15] Motoshima H, Wu X, Mahadev K, Goldstein BJ. Adiponectin suppresses proliferation and superoxide generation and enhances eNOS activity in endothelial cells treated with oxidized LDL. Biochem Biophys Res Commun. 2004;315(2):264-71. 10.1016/j.bbrc.2004.01.049 14766203

[16] Pineiro R, Iglesias MJ, Gallego R, Raghay K, Eiras S, Rubio J, et al. Adiponectin is synthesized and secreted by human and murine cardiomyocytes. FEBS Lett. 2005;579(23):5163-9. 10.1016/j.febslet.2005.07.098 16140297

[17] Kharroubi I, Rasschaert J, Eizirik DL, Cnop M. Expression of adiponectin receptors in pancreatic beta cells. Biochem Biophys Res Commun. 2003;312(4):1118-22. 10.1016/j.bbrc.2003.11.042 14651988

[18] Tan KC, Xu A, Chow WS, Lam MC, Ai VH, Tam SC, et al. Hypoadiponectinemia is associated with impaired endothelium-dependent vasodilation. J Clin Endocrinol Metab. 2004;89(2):765-9. 10.1210/jc.2003-031012 14764794

[19] Coope A, Milanski M, Araujo EP, Tambascia M, Saad MJ, Geloneze B, et al. AdipoR1 mediates the anorexigenic and insulin/leptin-like actions of adiponectin in the hypothalamus. FEBS letters. 2008;582(10):1471-6. 10.1016/j.febslet.2008.03.037 18394428

[20] Tsuchida A, Yamauchi T, Ito Y, Hada Y, Maki T, Takekawa S, et al. Insulin/Foxo1 pathway regulates expression levels of adiponectin receptors and adiponectin sensitivity. J Biol Chem. 2004;279(29):30817-22. 10.1074/jbc.M402367200 15123605

[21] Gil-Campos M, Canete RR, Gil A. Adiponectin, the missing link in insulin resistance and obesity. Clin Nutr. 2004;23(5):963-74. 10.1016/j.clnu.2004.04.010 15380884

[22] Heid IM, Wagner SA, Gohlke H, Iglseder B, Mueller JC, Cip P, et al. Genetic architecture of the APM1 gene and its influence on adiponectin plasma levels and parameters of the metabolic syndrome in 1,727 healthy Caucasians. Diabetes. 2006;55(2):375-84. 10.2337/diabetes.55.02.06.db05-0747 16443770

[23] Kern PA, Di Gregorio GB, Lu T, Rassouli N, Ranganathan G. Adiponectin expression from human adipose tissue: relation to obesity, insulin resistance, and tumor necrosis factor-alpha expression. Diabetes. 2003;52(7):1779-85. 10.2337/diabetes.52.7.1779 12829646

[24] Hara K, Boutin P, Mori Y, Tobe K, Dina C, Yasuda K, et al. Genetic variation in the gene encoding adiponectin is associated with an increased risk of type 2 diabetes in the Japanese population. Diabetes. 2002;51(2):536-40. 10.2337/diabetes.51.2.536 11812766

[25] Menzaghi C,Trischitta V, Doria A. Genetic influences of adiponectin on insulin resistance, type 2 diabetes, and cardiovascular disease. Diabetes. 2007;56(5):1198-209. 10.2337/db06-0506 17303804

[26] Schulze MB, Shai I, Rimm EB, Li T, Rifai N, Hu FB. Adiponectin and future coronary heart disease events among men with type 2 diabetes. Diabetes. 2005;54(2):534-9. 10.2337/diabetes.54.2.534 15677512

[27] Oliveira CS, Saddi-Rosa P, Crispim F, Canani LH, Gerchman F, Giuffrida FM, et al. Association of ADIPOQ variants, total and high molecular weight adiponectin levels with coronary artery disease in diabetic and non-diabetic Brazilian subjects. J Diabetes Complications. 2012;26(2):94-8. 10.1016/j.jdiacomp.2012.02.008 22459242

[28] Yamauchi T, Kamon J, Waki H, Terauchi Y, Kubota N, Hara K, et al. The fat-derived hormone adiponectin reverses insulin resistance associated with both lipoatrophy and obesity. Nat Med. 2001;7(8):941-6. 10.1038/90984 11479627

[29] Yamauchi T, Kamon J, Minokoshi Y, Ito Y, Waki H, Uchida S, et al. Adiponectin stimulates glucose utilization and fatty-acid oxidation by activating AMP-activated protein kinase. Nat Med. 2002;8(11):1288-95. 10.1038/nm788 12368907

[30] Berg AH, Combs TP, Du X, Brownlee M, Scherer PE. The adipocyte-secreted protein Acrp30 enhances hepatic insulin action. Nat Med. 2001;7(8):947-53. 10.1038/90992 11479628

[31] Combs TP, Berg AH, Obici S, Scherer PE, Rossetti L. Endogenous glucose production is inhibited by the adipose-derived protein Acrp30. J Clin Invest. 2001;108(12):1875-81. 10.1172/JCI14120 PMC209474 11748271

[32] Devaraj S, Glaser N, Griffen S, Wang-Polagruto J, Miguelino E, Jialal I. Increased monocytic activity and biomarkers of inflammation in patients with type 1 diabetes. Diabetes. 2006;55(3):774-9. 10.2337/diabetes.55.03.06.db05-1417 16505242

[33] Pradhan AD, Manson JE, Rifai N, Buring JE, Ridker PM. C-reactive protein, interleukin 6, and risk of developing type 2 diabetes mellitus. JAMA. 2001;286(3):327-34. 10.1001/jama.286.3.327 11466099

[34] Mills KT, Hamm LL, Alper AB, Miller C, Hudaihed A, Balamuthusamy S, et al. Circulating adipocytokines and chronic kidney disease. PloS One. 2013;8(10):e76902. 10.1371/journal.pone.0076902 PMC3792047 24116180

[35] Briffa JF, McAinch AJ, Poronnik P, Hryciw DH. Adipokines as a link between obesity and chronic kidney disease. Am J Physiol Renal Physiol. 2013;305(12):F1629-36. 10.1152/ajprenal.00263.2013 24107418

[36] Cha JJ, Min HS, Kim K, Lee MJ, Lee MH, Kim JE, et al. Long-term study of the association of adipokines and glucose variability with diabetic complications. Korean J Intern Med. 2016 Nov 4. doi: 10.3904/kjim.2016.114. 10.3904/kjim.2016.114 PMC5840591 27809453

[37] Jorsal A, Tarnow L, Frystyk J, Lajer M, Flyvbjerg A, Parving HH, et al. Serum adiponectin predicts all-cause mortality and end stage renal disease in patients with type I diabetes and diabetic nephropathy. Kidney Int. 2008;74(5):649-54. 10.1038/ki.2008.201 18496510

[38] Menon V, Li L, Wang X, Greene T, Balakrishnan V, Madero M, et al. Adiponectin and mortality in patients with chronic kidney disease. J Am Soc Nephrol. 2006;17(9):2599-606. 10.1681/ASN.2006040331 16885405

[39] Martinez Cantarin MP, Keith SW, Waldman SA, Falkner B. Adiponectin receptor and adiponectin signaling in human tissue among patients with end-stage renal disease. Nephrol Dial Transplant. 2014;29(12):2268-77 10.1093/ndt/gfu249 PMC4240178 25049200

[40] Martinez Cantarin MP, Waldman SA, Doria C, Frank AM, Maley WR, Ramirez CB, et al. The adipose tissue production of adiponectin is increased in end-stage renal disease. Kidney Int. 2013;83(3):487-94. 10.1038/ki.2012.421 PMC3587362 23283133

[41] Weijnen CF, Rich SS, Meigs JB, Krolewski AS, Warram JH. Risk of diabetes in siblings of index cases with Type 2 diabetes: implications for genetic studies. Diabet Med. 2002;19(1):41-50. 10.1046/j.1464-5491.2002.00624.x 11869302

[42] Keramati AR, Fathzadeh M, Go GW, Singh R, Choi M, Faramarzi S, et al. A form of the metabolic syndrome associated with mutations in DYRK1B. N Engl J Med. 2014;370(20):1909-19. 10.1056/NEJMoa1301824 PMC4069260 24827035

[43] Smemo S, Tena JJ, Kim KH, Gamazon ER, Sakabe NJ, Gomez-Marin C, et al. Obesity-associated variants within FTO form long-range functional connections with IRX3. Nature. 2014;507(7492):371-5. 10.1038/nature13138 PMC4113484 24646999

[44] Florez JC. Clinical review: the genetics of type 2 diabetes: a realistic appraisal in 2008. J Clin Endocrinol Metab. 2008;93(12):4633-42. 10.1210/jc.2008-1345 PMC2626447 18782870

[45] Comuzzie AG, Funahashi T, Sonnenberg G, Martin LJ, Jacob HJ, Black AE, et al. The genetic basis of plasma variation in adiponectin, a global endophenotype for obesity and the metabolic syndrome. J Clin Endocrinol Metab. 2001;86(9):4321-5. 10.1210/jcem.86.9.7878 11549668

[46] Lindsay RS, Funahashi T, Krakoff J, Matsuzawa Y, Tanaka S, Kobes S, et al. Genome-wide linkage analysis of serum adiponectin in the Pima Indian population. Diabetes. 2003;52(9):2419-25. 10.2337/diabetes.52.9.2419 12941784

[47] Okamoto Y, Kihara S, Funahashi T, Matsuzawa Y, Libby P Adiponectin: a key adipocytokine in metabolic syndrome. Clin Sci (Lond). 2006;110(3):267-78. 10.1042/CS20050182 16464169

[48] Vasseur F, Helbecque N, Dina C, Lobbens S, Delannoy V, Gaget S, et al. Single-nucleotide polymorphism haplotypes in the both proximal promoter and exon 3 of the APM1 gene modulate adipocyte-secreted adiponectin hormone levels and contribute to the genetic risk for type 2 diabetes in French Caucasians. Hum Mol Genet. 2002;11(21):2607-14. 10.1093/hmg/11.21.2607 12354786

[49] Stumvoll M, Tschritter O, Fritsche A, Staiger H, Renn W, Weisser M, et al. Association of the T-G polymorphism in adiponectin (exon 2) with obesity and insulin sensitivity: interaction with family history of type 2 diabetes. Diabetes. 2002;51(1):37-41. 10.2337/diabetes.51.1.37 11756320

[50] Fan Y. Association between ADIPOQ +45T>G Polymorphism and Type 2 Diabetes: A Systematic Review and Meta-Analysis. Int J Mol Sci. 2015;16:704-23. 10.3390/ijms16010704 PMC4307270 25561226

[51] Hivert MF, Sullivan LM, Shrader P, Fox CS, Nathan DM, D'Agostino RB Sr, et al. Insulin resistance influences the association of adiponectin levels with diabetes incidence in two population-based cohorts: the Cooperative Health Research in the Region of Augsburg (KORA) S4/F4 study and the Framingham Offspring Study. Diabetologia. 2011;54(5):1019-24. 10.1007/s00125-011-2067-y PMC3223124 21336532

[52] Lihn AS, Bruun JM, He G, Pedersen SB, Jensen PF, Richelsen B. Lower expression of adiponectin mRNA in visceral adipose tissue in lean and obese subjects. Mol Cell Endocrinol. 2004;219(1-2):9-15. 10.1016/j.mce.2004.03.002 15149722

[53] Fisher FM, McTernan PG, Valsamakis G, Chetty R, Harte AL, Anwar AJ, et al. Differences in adiponectin protein expression: effect of fat depots and type 2 diabetic status. Horm Metab Res. 2002;34(11-12):650-4. 10.1055/s-2002-38246 12660876

[54] Cnop M, Havel PJ, Utzschneider KM, Carr DB, Sinha MK, Boyko EJ, et al. Relationship of adiponectin to body fat distribution, insulin sensitivity and plasma lipoproteins: evidence for independent roles of age and sex. Diabetologia. 2003;46(4):459-69. 10.1007/s00125-003-1074-z 12687327

[55] Arita Y, Kihara S, Ouchi N,Takahashi M, Maeda K, Miyagawa J, et al. Paradoxical decrease of an adipose-specific protein, adiponectin, in obesity. Biochem Biophys Res Commun. 1999;257(1):79-83. 10.1006/bbrc.1999.0255 10092513

[56] Daimon M, Oizumi T, Saitoh T, Kameda W, Hirata A, Yamaguchi H, et al. Decreased serum levels of adiponectin are a risk factor for the progression to type 2 diabetes in the Japanese Population: the Funagata study. Diabetes Care. 2003;26(7):2015-20. 10.2337/diacare.26.7.2015 12832305

[57] Iwashima Y, Katsuya T, Ishikawa K, Ouchi N, Ohishi M, Sugimoto K, et al. Hypoadiponectinemia is an independent risk factor for hypertension. Hypertension. 2004;43(6):1318-23. 10.1161/01.HYP.0000129281.03801.4b 15123570

[58] Matsubara M, Maruoka S, Katayose S. Decreased plasma adiponectin concentrations in women with dyslipidemia. J Clin Endocrinol Metab. 2002;87(6):2764-9. 10.1210/jcem.87.6.8550 12050247

[59] Liao D, Arnett DK, Tyroler HA, Riley WA, Chambless LE, Szklo M, et al. Arterial stiffness and the development of hypertension. The ARIC study. Hypertension. 1999;34(2):201-6. 10.1161/01.hyp.34.2.201 10454441

[60] Schillaci G, Pirro M, Vaudo G, Mannarino MR, Savarese G, Pucci G, et al. Metabolic syndrome is associated with aortic stiffness in untreated essential hypertension. Hypertension. 2005;45(6):1078-82. 10.1161/01.HYP.0000165313.84007.7d 15867139

[61] Tsai WC, Lin CC, Chen JY, Huang YY, Lee CH, Li WT, et al. Association of adiponectin with procollagen type I carboxyterminal propeptide in non-diabetic essential hypertension. Blood Press. 2008;17(4):233-8. 10.1080/08037050802308895 18671144

[62] Snijder MB, Flyvbjerg A, Stehouwer CD, Frystyk J, Henry RM, Seidell JC, et al. Relationship of adiposity with arterial stiffness as mediated by adiponectin in older men and women: the Hoorn Study. Eur J Endocrinol. 2009;160(3):387-95. 10.1530/EJE-08-0817 19095778

[63] Vaiopoulos AG, Marinou K, Christodoulides C, Koutsilieris M. The role of adiponectin in human vascular physiology. Int J Cardiol. 2012;155(2):188-93. 10.1016/j.ijcard.2011.07.047 21907426

[64] Xu A, Vanhoutte PM. Adiponectin and adipocyte fatty acid binding protein in the pathogenesis of cardiovascular disease. Am J Physiol Heart Circ Physiol. 2012;302(6):H1231-40. 10.1152/ajpheart.00765.2011 22210749

[65] Brasier AR, Recinos A, 3rd, Eledrisi MS. Vascular inflammation and the renin-angiotensin system. Arterioscler Thromb Vasc Biol. 2002;22(8):1257-66. 10.1161/01.atv.0000021412.56621.a2 12171785

[66] de Kloet AD, Krause EG, Woods SC. The renin angiotensin system and the metabolic syndrome. Physiol Behav. 2010;100(5):525-34. 10.1016/j.physbeh.2010.03.018 PMC2886177 20381510

[67] van Stijn CM, Kim J, Barish GD, Tietge UJ, Tangirala RK. Adiponectin expression protects against angiotensin II-mediated inflammation and accelerated atherosclerosis. PloS One. 2014;9(1):e86404. 10.1371/journal.pone.0086404 PMC3899253 24466075

[68] Laughlin GA, Barrett-Connor E, May S, Langenberg C. Association of adiponectin with coronary heart disease and mortality: the Rancho Bernardo study. Am J Epidemiol. 2007;165(2):164-74. 10.1093/aje/kwk001 PMC2642645 17101706

[69] Koh SB, Yoon J, Kim JY, Yoo BS, Lee SH, Park JK, et al. Relationships between serum adiponectin with metabolic syndrome and components of metabolic syndrome in non-diabetic Koreans: ARIRANG study. Yonsei Med J. 2011;52(2):234-41. 10.3349/ymj.2011.52.2.234 PMC3051212 21319340

[70] von Frankenberg AD, do Nascimento FV, Gatelli LE, Nedel BL, Garcia SP, de Oliveira CS, et al. Major components of metabolic syndrome and adiponectin levels: a cross-sectional study. Diabetol Metab Syndr. 2014;6(1):26. 10.1186/1758-5996-6-26 PMC3941563 24568287

[71] Mantzoros CS, Williams CJ, Manson JE, Meigs JB, Hu FB. Adherence to the Mediterranean dietary pattern is positively associated with plasma adiponectin concentrations in diabetic women. Am J Clin Nutr. 2006;84(2):328-35. 10.1093/ajcn/84.1.328 16895879

[72] von Frankenberg AD, Silva FM, de Almeida JC, Piccoli V, do Nascimento FV, Sost MM, et al. Effect of dietary lipids on circulating adiponectin: a systematic review with meta-analysis of randomised controlled trials. Br J Nutr. 2014;112(8):1235-50. 10.1017/S0007114514002013 25192422

[73] Arvidsson E, Viguerie N, Andersson I, Verdich C, Langin D, Arner P. Effects of different hypocaloric diets on protein secretion from adipose tissue of obese women. Diabetes. 2004;53(8):1966-71. 10.2337/diabetes.53.8.1966 15277374

[74] Marina A, von Frankenberg AD, Suvag S, Callahan HS, Kratz M, Richards TL, et al. Effects of dietary fat and saturated fat content on liver fat and markers of oxidative stress in overweight/obese men and women under weight-stable conditions. Nutrients. 2014;6(11):4678-90. 10.3390/nu6114678 PMC4245556 25353663

[75] Wycherley TP, Brinkworth GD, Keogh JB, Noakes M, Buckley JD, Clifton PM. Long-term effects of weight loss with a very low carbohydrate and low fat diet on vascular function in overweight and obese patients. J Intern Med. 2010;267(5):452-61. 10.1111/j.1365-2796.2009.02174.x 20141567

[76] Trichopoulou A, Costacou T, Bamia C, Trichopoulos D. Adherence to a Mediterranean diet and survival in a Greek population. N Engl J Med. 2003;348(26):2599-608. 10.1056/NEJMoa025039 12826634

[77] Neschen S, Morino K, Rossbacher JC, Pongratz RL, Cline GW, Sono S, et al. Fish oil regulates adiponectin secretion by a peroxisome proliferator-activated receptor-gamma-dependent mechanism in mice. Diabetes. 2006;55(4):924-8. 10.2337/diabetes.55.04.06.db05-0985 16567512

[78] Yamauchi T, Kadowaki T Physiological and pathophysiological roles of adiponectin and adiponectin receptors in the integrated regulation of metabolic and cardiovascular diseases. Int J Obes (Lond). 2008;32 Suppl 7:S13-8. 10.1038/ijo.2008.233 19136982

[79] Tsuchida A, Yamauchi T, Takekawa S, Hada Y, Ito Y, Maki T, et al. Peroxisome proliferator-activated receptor (PPAR)alpha activation increases adiponectin receptors and reduces obesity-related inflammation in adipose tissue: comparison of activation of PPARalpha, PPARgamma, and their combination. Diabetes. 2005;54(12):3358-70. 10.2337/diabetes.54.12.3358 16306350

[80] Martinez K, Kennedy A,West T, Milatovic D, Aschner M, McIntosh M. trans-10,cis-12-Conjugated linoleic acid instigates inflammation in human adipocytes compared with preadipocytes. J Biol Chem. 2010;285(23):17701-12. 10.1074/jbc.M109.043976 PMC2878534 20353947

[81] Poirier H, Shapiro JS, Kim RJ, Lazar MA. Nutritional supplementation with trans-10, cis-12-conjugated linoleic acid induces inflammation of white adipose tissue. Diabetes. 2006;55(6):1634-41. 10.2337/db06-0036 16731825

[82] Brown JM, Boysen MS, Chung S, Fabiyi O, Morrison RF, Mandrup S, et al. Conjugated linoleic acid induces human adipocyte delipidation: autocrine/paracrine regulation of MEK/ERK signaling by adipocytokines. J Biol Chem. 2004;279(25):26735-47. 10.1074/jbc.M401766200 PMC1351018 15067015

[83] Iwaki M, Matsuda M, Maeda N, Funahashi T, Matsuzawa Y, Makishima M, et al. Induction of adiponectin, a fat-derived antidiabetic and antiatherogenic factor, by nuclear receptors. Diabetes. 2003;52(7):1655-63. 10.2337/diabetes.52.7.1655 12829629

